# Motivational Coaching Cues Can Modulate Prefrontal Cortex Activity and Perceptual Responses of Elite Football Players During High‐Intensity Interval Training: A Randomised Crossover fNIRS Study

**DOI:** 10.1002/ejsc.70222

**Published:** 2026-07-30

**Authors:** Sanghyuk Han, SoYoung Ahn, Yunho Sung, Ji‐won Seo, Xinxing Li, Seungoh Han, Chaewoon Kim, Wook Song

**Affiliations:** ^1^ Department of Physical Education Seoul National University Seoul Republic of Korea; ^2^ College of Wushu and Dance Shenyang Sport University Shenyang China; ^3^ Institute of Sport Science Seoul National University Seoul Republic of Korea; ^4^ Institute on Aging Seoul National University Seoul Republic of Korea

**Keywords:** fNIRS, high‐intensity interval training, motivation, neural efficiency, prefrontal cortex

## Abstract

Football coaches commonly provide verbal encouragement and exercise end‐point feedback to sustain players' effort by enhancing motivation during fitness training such as high‐intensity interval training (HIIT). However, the influence of such cues on the prefrontal cortex (PFC), a region involved in exercise cessation decisions, remains unclear. Therefore, this study aimed to compare the effect of motivational cues on PFC activation during HIIT using functional near‐infrared spectroscopy, alongside physiological and perceptual responses. Twenty‐two male elite footballers completed two randomised crossover HIIT sessions on a cycle ergometer, with and without motivational cues. Each session consisted of 12 repetitions of 30‐s high‐intensity bouts, each interspersed with a 30‐s active recovery bout. Heart rate, lactate concentration, rating of perceived exertion (RPE), motivation‐related questionnaires and oxygenated haemoglobin (HbO_2_) concentration in the PFC were measured throughout HIIT. Although physiological responses were similar between conditions, perceived difficulty, assessed every three HIIT bouts, was significantly lower under the motivational condition, with lower RPE (*p* = 0.038) and higher motivation (*p* = 0.049), engagement (*p* = 0.038) and mood (*p* = 0.009) scores, particularly after the 9th repetition. From the 10th to 12th repetition of HIIT, the HbO_2_ concentrations of the orbitofrontal cortex and frontopolar PFC were significantly lower under the motivational condition (*p* = 0.040 and *p* = 0.036, respectively), whereas the dorsolateral and ventrolateral PFC showed no significant difference between conditions. In conclusion, motivational cues reduce central PFC activity during the most fatiguing phases of HIIT, reflecting enhanced neural efficiency under motivation despite equivalent physical workloads.

## Highlights

- Motivational coaching cues lead to lower perceptual difficulties and reduced central PFC demand during the later phase of HIIT when fatigue is most pronounced, indicating greater neural efficiency despite equivalent workloads.
- Differences in the central and lateral PFC responses may reflect region‐specific functional roles in the neural processing of motivational stimuli during repeated and fatiguing exercise.
- Coaches can employ motivational cues as a match strategy to delay performance impairment from prolonged physical and cognitive loads by attenuating central PFC activation and perceived effort.

## 1 Introduction

During a football match, while most distance is covered at lower speeds, roughly 22%–24% occurs above 15 km/h, 8%–9% above 20 km/h, and 2%–3% above 25 km/h, with players executing between 600 and 650 accelerations throughout (Rampinini et al. 2007). These high‐intensity demands have increased in recent years, with high‐speed running and sprint distances rising by 30%–35% between the 2006/07 and the 2012/13 season in the English Premier League despite similar total distance covered (Barnes et al. 2014). Alongside the greater physical demands and higher tempo, football players are constantly required to cognitively anticipate and react in a changing and unpredictable environment under opponent pressure with limited time (Pruna and Bahdur 2016). Prolonged exposure to cognitive loads can induce a psychobiological state of mental fatigue accompanied by subjective feelings of tiredness and lack of energy (Marcora et al. 2009; Nedelec et al. 2012). The accumulated mental fatigue can provoke endurance performance impairment (Marcora et al. 2009; Van Cutsem et al. 2017) alongside more technical errors towards the end of a match, which may directly impact the score (Russell et al. 2011). An analysis of 1881 goals scored across the previous 14 FIFA (Federation Internationale de Football Association) World Cups (1966–2018) revealed that 56.3% of goals occurred in the second half of matches and the time period between the 76th minute and the final whistle had the highest scoring rate, accounting for 22.7% of all goals (Micovic et al. 2023). Accordingly, it is essential for footballers to have a competitive aerobic capacity (Dolci et al. 2020) through fitness training such as high‐intensity interval training (HIIT) which is a well‐known time‐efficient training method (Costigan et al. 2015) and popular among endurance athletes and team sports (Thomakos et al. 2024; Engel et al. 2018; Zuniga et al. 2011).

In order to develop aerobic capacity, identifying the factors that limit time to exhaustion is crucial (Gandevia 2001). There has been a long‐standing assumption that the cessation of high‐intensity aerobic exercise is primarily caused by a failure of producing required power output due to fatigue in the locomotor muscles (Allen et al. 2008). However, from a psychobiological perspective, it was proposed that cessation of high‐intensity aerobic exercise results from a conscious decision‐making process in which perception of effort plays a central role (Staiano et al. 2018). Any physiological or psychological factor that alters perception of effort has the potential to affect endurance performance even if locomotor muscle fatigue is not influenced (Pageaux et al. 2015). For example, motivational factors can interact with perception of effort to determine willingness to sustain aerobic exercise (Wright 2008). Motivational stimuli such as verbal instruction and encouragement are commonly adopted in sports training to achieve better results (Halperin et al. 2015). According to a systematic literature review of psychological interventions on endurance performance, verbal encouragement can have a beneficial effect on endurance performance (McCormick et al. 2015). Encouragement interventions during football training also affect mental and internal physical loads such as performance satisfaction, mental effort, rating of perceived exertion (RPE) and unsafety values (Díaz‐García et al. 2021). Similarly, exercise end‐point feedback such as exercise duration is important in controlling physiological and psychological resources during exercise (Ansley et al. 2004). Expectation of task duration and anticipation of an end‐point have been suggested as significant influence factors affecting RPE and associative thought processes (Baden et al. 2004; Baden et al. 2005).

The prefrontal cortex (PFC) activity appears to play a key role in successful endurance performance (Tucker and Noakes 2009) by mediating decisions to terminate exercise (De Wachter et al. 2021). The PFC, located in front of the neocortex, is involved in executive function which is the ability to select appropriate actions to achieve a certain goal (Miyake et al. 2000). The processing of executive functions is linked to the neural activity in the PFC (Cutini et al. 2012), which regulates regional blood flow to meet the metabolic demands, thereby altering oxygenated haemoglobin (HbO_2_) concentration (Heeger and Ress 2002). While PFC oxygenation is considered a potential limiting factor during exercise (Billaut et al. 2010), its neural underpinnings remain poorly understood, particularly in relation to motivation, which is a key component in exercise (Doren et al. 2024). To date, the effect of motivation on performance has mainly been examined using subjective questionnaires or interviews (Brunet and Sabiston 2011). To provide a more objective assessment from a neurobiological perspective, changes in brain activity were investigated using functional near‐infrared spectroscopy (fNIRS) during periods of additional motivation in maximal speed walking (Doren et al. 2024). Their findings revealed that healthy adults exhibited increased PFC activation when walking under extra motivation conditions including verbal encouragement and auditory count of the lap time, compared to trials without such motivational stimuli. However, the effect of motivational cues on the PFC activity during more fatiguing exercise where athletes rely heavily on motivation to sustain performance has not yet been examined. Therefore, the present study aimed to examine the impact of coaching cues intended to enhance motivation on changes in PFC oxygenation in elite football players during HIIT using fNIRS, alongside physiological and perceptual responses. Based on the aforementioned evidence, it was hypothesised that coaching cues to motivate players during the most fatiguing phases of HIIT would result in significantly different changes in HbO_2_ concentration in the PFC as well as lower perceived difficulties, compared to the control condition.

## 2 Materials and Methods

### 2.1 Participants

A minimum sample size of 22 was determined using G*Power version 3.1.9.4 software with an effect size (*d* = 0.8; Cohen 1988) consistent with comparable elite football research (Doren et al. 2024), power (1–β) of 0.95, and an alpha level of 0.05 (Faul et al. 2009). Considering a 15% dropout rate, a total of 25 male elite football players were recruited. Participants were required to meet the following inclusion criteria: (a) a male football player with a minimum of 6 years of competitive football training experience; (b) aged between 19 and 29 years; (c) no history of any major disease; (d) no history of surgeries within 5 years due to injury; (e) non‐smoker and non‐drug user; and (f) no difficulties using a cycle ergometer. As this study was designed as a single‐blind trial, information regarding motivational intervention was intentionally excluded during the explanation of the experiment to minimise expectancy effects and prevent potential bias in participant responses. All participants provided written informed consent prior to the experiment participation. This study was approved by the Institutional Review Board of Seoul National University, Seoul, Republic of Korea (IRB approval no. 2412/002–021). The experiment followed the principles of the Declaration of Helsinki.

### 2.2 Procedure

**FIGURE 1:**
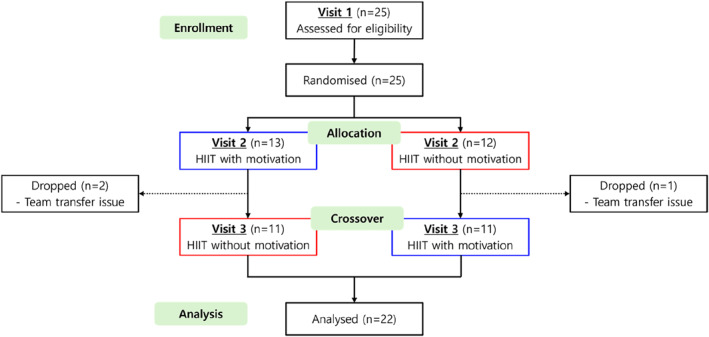
Flowchart of a randomised and counterbalanced crossover study in elite football players. HIIT, high‐intensity interval training.

The schematic of the experimental design is illustrated in Figure 1. This study was a randomised, counterbalanced crossover design. Participants were required to attend on three occasions with an interval of at least 7 days between the visits to prevent carryover effects and were instructed to avoid vigorous physical activity and alcohol. Caffeine intake was also restricted for 24 hours before each visit to ensure complete metabolic clearance, preventing its stimulatory effects from confounding baseline physiological measurements. Habitual dietary routines, including supplementation, were maintained to ensure that the research protocol did not compromise the athletes' ongoing training adaptations and recovery.

### 2.3 Visit 1

At the initial visit, the participants underwent screening measures for the inclusion criteria and performed a maximal graded exercise test. This test was performed on an electromagnetically braked cycle ergometer (Corival CPET, Lode, the Netherlands) to establish maximal oxygen uptake (VO_2_max) and peak power output (PPO), and the cardiorespiratory capacity was assessed using a gas analyser (Quark CPET, COSMED, Italy). After a 5‐min low‐intensity warm‐up, the graded exercise test began at 100 W for 2 minutes, after which the power output was increased in increments of 30 W every 2 minutes until exhaustion. The high initial resistance (100 W) was implemented to help highly fit subjects reach VO_2_max within the widely recommended 8–12‐min duration (Buchfuhrer et al. 1983). During the entire test, the participants were required to maintain a constant speed of 60 revolutions per minute (rpm). The test was terminated when each participant expressed their inability to continue due to increasing resistance or could no longer maintain 60 rpm despite strong verbal encouragement while the participants met at least two of the following criteria (Day et al. 2003): (a) VO_2_ plateau, (b) heart rate > 90% of age‐predicted maximal heart rate (220‐age) (Fox et al. 1971), and (c) respiratory exchange ratio > 1.20. The PPO recorded during the test was used for the individualised intensity of the HIIT protocol in subsequent visits.

### 2.4 Visit 2 and 3

A total of 25 participants were successfully enrolled and randomly allocated into two groups. During the 2nd visit, one group (*n* = 13) completed a HIIT session with motivation, while the other group (*n* = 12) completed a HIIT session without motivation. In the 3rd visit, the participants crossed over to perform a HIIT session under the opposite condition. Three participants dropped out due to team transfer issues, resulting in a final sample of 22 participants (*n* = 11 for each group) included in the analysis. In the following two separate visits, participants performed the same HIIT session using the cycle ergometer under either training environment: with or without motivational coaching cues. The HIIT session consisted of twelve 30‐s bouts at 100% PPO, each interspersed with 30‐s active recovery periods at 35% PPO during which participants were required to maintain a pedal cadence over 60 rpm. Upon completing the session, the participants were allowed to cool down for as long as they wanted.

### 2.5 Motivational Coaching Cues

For the motivational intervention, concurrent verbal encouragements such as “Great effort!”, “Keep pushing!” and “Don't give up!” were constantly delivered by the examiner over the high‐intensity bout. Moreover, auditory countdowns indicating the remaining exercise time were provided at the end of each high‐intensity bout, using phrases like “5 seconds to go!” or “Three, two, one, and slow down.”. Furthermore, once the participants had completed half of the total session volume, the examiner offered progress updates about the remaining repetitions to support goal‐directed effort. For example, at the beginning of either a high‐intensity or recovery bout, statements such as “Only three repetitions left!” or “Once you complete this repetition, you will have finished two‐thirds!” were announced. In the control condition, none of these additional stimuli were presented during the protocol and participants performed the HIIT session without being informed of the target exercise volume (12 repetitions). The examiner only provided notifications of the transitions in exercise intensity, delivered in a calm tone of voice.

### 2.6 Measurements

On the 1st visit, descriptive data of the participants were collected and the inclusion criteria were assessed by a medical health/history questionnaire. Body composition assessment was conducted using a bioelectrical impedance analyser (InBody 770, InBody Co. Ltd., South Korea). Weekly physical activity was also assessed using the International Physical Activity Questionnaire (IPAQ) to monitor variations in physical activity between the visits such as training volume. During the HIIT session on the 2nd and 3rd visit, heart rate was monitored using a chest‐strap device (Polar Team2, Polar, Finland) over the entire period of the protocols and blood lactate concentration was measured via a portable analyser (Lactate Pro 2 LT‐1730, ARKRAY, Japan) at rest and immediately after the final intense bout. In addition, the Borg RPE scale (6–20) was recorded at the end of every three repetitions (3rd, 6th, 9th, and 12th) of the intense bout to assess the individual's perceived effort. Participants assessed their levels of motivation, engagement, and mood using a 7‐point bipolar Likert scale, ranging from “extremely unlikely/negative” to “extremely likely/positive”. They answered the following questions: “How motivated are you right now to complete the remaining exercise?”, “How willing are you right now to engage in the remaining exercise?”, “How is your mood right now?” to evaluate the effect of the motivational intervention provided over the HIIT session. These scores were recorded before and after the 3rd, 6th, and 9th repetition of HIIT.

### 2.7 Functional Near‐Infrared Spectroscopy (fNIRS)

**FIGURE 2:**
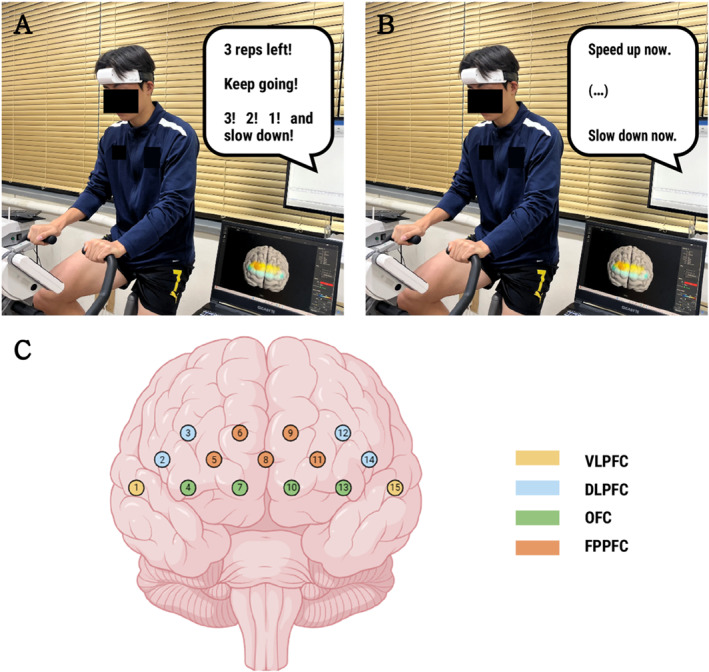
Measurement of prefrontal cortex (PFC) via functional near‐infrared spectroscopy (fNIRS). Scenes of measuring PFC oxygenation during high‐intensity interval training under motivational (A) and control condition (B). Location of PFC regions by channel (C). 1 and 15, ventrolateral prefrontal cortex (VLPFC); 2, 3, 12, and 14, dorsolateral prefrontal cortex (DLPFC); 4, 7, 10, and 13, orbitofrontal cortex (OFC); 5, 6, 8, 9, and 11, frontopolar prefrontal cortex (FPPFC).

Figure 2A and 2B show fNIRS measurements conducted during HIIT under two different motivational conditions. In this study, an fNIRS device (NIRSIT Lite, OBELAB Inc. Republic of Korea) was used to measure haemodynamic responses in the PFC by detecting changes in HbO_2_ concentration in the brain. This ultra‐lightweight (200 g), wearable and wireless device consists of 15 channels, each separated by a distance of 30 mm between pairs. Among these, channels 1–7 are located on the right PFC, channel 8 is positioned at the centre, and channels 9–15 are arranged symmetrically on the left PFC. The channels are further divided into four regions (Figure 2C): ventrolateral PFC (channels 1 and 15; VLPFC), dorsolateral PFC (channels 2, 3, 12, and 14; DLPFC), Frontopolar PFC (channels 5, 6, 8, 9, and 11; FPPFC), and orbitofrontal cortex (channels 4, 7, 10, and 13; OFC). The device, equipped with five dual‐wavelength laser sources (780/850 nm) and seven detectors, measures light diffusion within a tissue volume based on the light propagation model. With a source‐detector separation of 30 mm, the light can reach approximately 8 mm into the cortical surface. Channels with a low‐intensity threshold of less than 30 A.U. were rejected and replaced using the mean of nearby channels, with nearest‐neighbour interpolation applied for up to five consecutive invalid data points. The raw optical density signals were converted into changes in HbO_2_ concentration using the modified Beer–Lambert law, with extinction coefficients derived from Moaveni's calculation and no pathlength factor applied. Motion artefact correction was applied to the optical density signal using the temporal derivative distribution repair algorithm. To remove physiologically irrelevant effects, DCT‐based lowpass filtering with a cutoff frequency of 0.8 Hz was applied to the time‐series data prior to analysis. The baseline correction was defined as the signal from 0.2 seconds after the onset of each exercise bout and the temporal means of changes in HbO_2_ concentration in each channel were calculated by averaging the fNIRS data from the exercise start (0.2‐s) to the exercise end (30‐s) of each bout. The present study primarily reported HbO_2_ signals as they represent the most widely used indicator of cerebral cortical activity in fNIRS research (Leff et al. 2011; Vitorio et al. 2017). The HbO_2_ value generally demonstrates stronger associations with BOLD signals in fMRI, a higher signal‐to‐noise, and greater sensitivity to task‐induced neural activity compared to deoxygenated haemoglobin (HbR) (Gagnon et al. 2012; Huppert et al. 2006; Issa et al. 2016; Kinder et al. 2022). The HbR data are provided in Supporting Information S1: Figure S3 in Appendix B to validate that the observed cortical activation changes reflect genuine neural activity rather than vascular artefact (Kinder et al. 2022). The present study reported the fNIRS data by four different regions (OFC, FPPFC, VLPFC, and DLPFC) and it included only the high‐intensity bouts. This is because it is important to monitor the effect of motivation on changes in cortical activity when greater physical and cognitive demands are present. Moreover, to explore the overall pattern of cortical responses to progressively accumulating physical and psychological fatigue, the 12 time points were consolidated into four broader time intervals in a temporal order, rather than analysed individually.

### 2.8 Statistical Analysis

All data were analysed using SPSS version 29.0 (SPSS, IBM Corporation, USA). Data were expressed as the mean ± standard deviation or standard errors of difference. The Shapiro‐Wilk test was performed to assess the normality of data. Differences between the motivational conditions at each time point or interval were assessed using a paired *t*‐test for parametric data or a Wilcoxon Signed‐rank test for non‐parametric data. Moreover, changes in variables over time were analysed using either a one‐way repeated measures analysis of variance or the Friedman test depending on the distribution. Pairwise comparisons were employed to examine differences between conditions at specific time intervals of interest, rather than to assess trends over time. One‐tailed tests were applied for perceptual responses given the strong a priori directional assumptions supported by existing literature, while two‐tailed tests were adopted for cortical responses where directional expectations were less certain. Post‐hoc test analysis was performed using Bonferroni correction if significant results were found. Statistical significance was set at *p* < 0.05.

## 3 Results

The characteristics of the 22 participants who completed three visits are presented in Table 1. On each visit, the participants reported their weekly physical activity volumes to assess the change in their total training volumes via IPAQ and there was no significant difference in total weekly physical activity volumes between the intervals.

**TABLE 1:** Descriptives of participants (*n* = 22).

Characteristic	Mean (SD)
Age (years)	20.9 (1.7)
Height (cm)	176.9 (5.6)
Body mass (kg)	73.3 (5.4)
Body fat percentage (%)	13.0 (2.8)
Football experience (years)	11.0 (2.1)
IPAQ total physical activity (MET‐min/week)	
1st visit_…	5610.5 (1291.7)
2nd visit_…	5414.5 (1210.8)
3rd visit_…	5462.4 (1184.4)
VO_2_max (mL/kg/min)	48.3 (5.6)
Peak power output (watts)	293.6 (27.4)

Abbreviations: IPAQ, international physical activity questionnaire; MET, metabolic equivalent of task; SD, standard deviation; VO_2_max, maximal oxygen uptake.

### 3.1 Physiological Responses

Heart rate and blood lactate concentration significantly increased over time in both conditions (all *p* < 0.001). However, there was no significant difference between motivational conditions at all time points measured in both cardiac and metabolic responses (Supporting Information S1: Figure S1 and S2 in Appendix A for the details of the physiological responses).

### 3.2 Perceptual Responses

**FIGURE 3:**
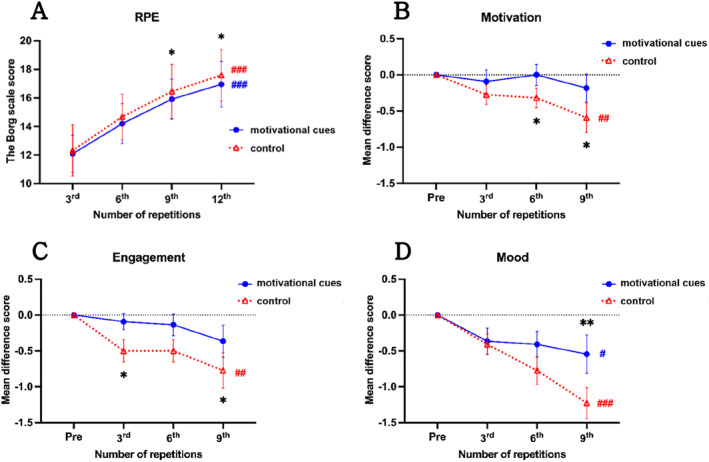
Changes in perceptual responses under different motivational conditions at four time points over high‐intensity interval training. Rating of perceived exertion (RPE) (A) and the scores of motivation‐related questionnaires about motivation (B), engagement (C), and mood (D). Error bars represent standard deviations in (A) or standard errors of difference in (B), (C), and (D). *p < 0.05, **p < 0.01 significantly higher in (A) or significantly lower in (B), (C), and (D) under the control condition compared to the motivational condition. #*p* < 0.05, ##*p* < 0.01, ###*p* < 0.001 significant increase over time for (A) or significant decrease over time for (B), (C), and (D).

The trend of RPE changes recorded every three repetitions throughout the HIIT sessions is shown in Figure 3A. It shows the significant increase over exercise time from start to finish in the subjective fatigue scores, regardless of motivational intervention (*p* < 0.001 for all conditions). With respect to the effect of motivation on RPE, no significant differences in RPE were found between the motivational conditions during the first half of the HIIT session. However, in the latter half of the total HIIT session, RPE scores under the control condition were significantly higher than those under the motivational condition (the 9th repetition, *p* = 0.038; the 12th repetition, *p* = 0.015). Figure 3B, 3C, and 3D present responses on a 7‐point bipolar Likert scale assessing three motivation‐related categories (motivation, engagement, and mood, respectively) measured at four time points (pre‐exercise, after the 3rd, 6th, and 9th repetition of high‐intensity bout). Scores in all categories recorded during the HIIT session in the control condition significantly decreased over time from the first to the final time point measured (motivation, *p* = 0.006; engagement, *p* = 0.004; mood, *p* < 0.001). In contrast, under the motivational condition, such a significant decrease was observed only in the mood category (*p* = 0.032). After the 9th repetition of high‐intensity exercise, the mean difference scores of all categories were significantly lower in the control condition compared to the motivational condition (motivation, *p* = 0.049; engagement, *p* = 0.038; mood, *p* = 0.009). Additionally, significantly lower scores were also observed in the earlier bouts of exercise, for example, in the engagement category after the 3rd repetition (*p* = 0.030) and in the motivation category after the 6th repetition (*p* = 0.044).

### 3.3 Cortical Responses

**FIGURE 4:**
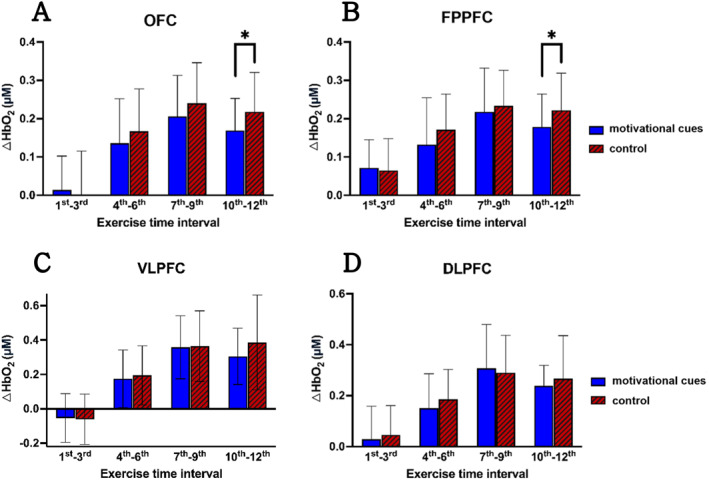
Average changes in oxygenated haemoglobin concentration (△HbO_2_) over four time intervals (1st–3rd, 4th–6th, 7th–9th, and 10th–12th) according to four regions (OFC, FPPFC, VLPFC, and DLPFC). Error bars represent standard deviations. OFC, orbitofrontal cortex (A); FPPFC, frontopolar prefrontal cortex (B); VLPFC, ventrolateral prefrontal cortex (C); DLPFC, dorsolateral prefrontal cortex (D). *p < 0.05 significant difference between the conditions.

Figure 4 indicates the average changes in HbO_2_ concentration in the four different regions (OFC, FPPFC, VLPFC, and DLPFC). In OFC, there was no significant difference of HbO_2_ concentration changes between the conditions during the early to middle phases of HIIT (the 1st–9th repetition). However, during the final interval (the 10th–12th repetition), the level of OFC oxygenation was significantly lower in the motivational condition compared to the control condition (*p* = 0.040). The difference of FPPFC oxygenation level between the conditions was not significant during the early to middle phases of HIIT (the 1st–9th repetition). However, during the final interval (the 10th–12th repetition), average changes in HbO_2_ concentration in the FPPFC were significantly lower in the motivational condition compared to the control condition (*p* = 0.036). In the VLPFC and DLPFC, no significant differences in HbO_2_ concentration changes between the conditions were found at any time interval of HIIT measured.

## 4 Discussion

This study investigated the effects of motivational coaching cues on physiological, perceptual, and cortical responses of elite football players during HIIT. The main findings indicate that physiological responses (heart rate and blood lactate concentration) did not significantly differ between the motivational conditions, whereas perceptual (RPE and motivation‐related questionnaires) and cortical responses (HbO_2_ concentration) were significantly different between the conditions. More specifically, during the final time interval (10th–12th) of HIIT when the difference in perceptual difficulty between conditions became significant, the average change in HbO_2_ concentration of the two central PFC regions (OFC and FPPFC) was significantly lower under the motivational condition, compared to the control condition. In contrast, PFC oxygenation in the lateral regions (VLPFC and DLPFC) did not show significant differences between the conditions at any time intervals measured during HIIT.

The impact of motivational cues on PFC oxygenation varied across different time intervals of the high‐intensity exercise session. During the earlier exercise bouts (1st–9th), no significant differences were observed between motivational and control conditions across any PFC regions. However, during the final exercise bouts (10th–12th), the motivational intervention led to significantly lower changes in HbO_2_ concentration specifically in the central PFC regions. This temporal pattern coincided with the time point (after the 9th repetition) when the RPE began to show a significant difference between motivational conditions, and when all three motivation‐related scores became significantly lower under the control condition compared to the motivational condition. It suggests that motivational effects on brain activity may become more pronounced as physical and mental fatigue progressively accumulate. Moreover, fatigue induced by physical and mental exertion, which alters perception of effort, negatively affects subsequent endurance performance (Pageaux and Lepers 2016). In line with this, the present study demonstrated that motivational interventions significantly altered RPE when physical and cognitive loads accumulated, enabling the participants to complete an equivalent physical task at different levels of perceived fatigue. This efficient task performance may be facilitated by modulations in HbO_2_ concentration and the neural demands required to sustain performance under fatigue. Consistent with the present findings, coach encouragement during football practices significantly influences players' mental and perceptual loads, suggesting that motivational coaching cues are a potent driver of RPE and motivation‐related outcomes (Díaz‐García et al. 2021). However, while the present study observed a reduction in RPE during HIIT, Díaz‐García et al. (2021) reported an increase during small‐sided games. This discrepancy may reflect differing training demands, as motivational cues in effort‐maximising contexts such as small‐sided games may elevate RPE, whereas in HIIT, where resisting fatigue is the primary goal, they may instead attenuate perceived exertion by reinforcing perceptual resilience.

The reduced oxygenation in the OFC and FPPFC under the motivational condition during the later stages of exercise may be consistent with the neural efficiency theory, as supported by the HbR data (Supporting Information S1: Figure S3 in Appendix B), showing an inverse relationship with HbO_2_—an increase in HbO_2_ accompanied by a concurrent decrease in HbR—indicating that the observed blood flow changes reflect neural activation rather than vascular dysfunction (Kinder et al. 2022). In general, neural efficiency refers to lower energy expenditure in completing the same performance as well as better performance during the repetition of a task (Babiloni et al. 2009; Zhang et al. 2019). It is assumed that a lower activity change in the PFC results from improved automated neural processes (Schmaderer et al. 2023). For example, semi‐professional football players showed lower PFC activity during football‐specific cognitive tasks including familiar stimuli compared to the brain activity during general cognitive tasks including novel stimuli (Schmaderer et al. 2023). The experts recognised repetitive stimuli from their own experiences, and they accomplished the task goal with reduced cortical activation (Li and Smith 2021). In the context of the present study, during the same physical task, motivational cues resulted in lower cortical activity in the central regions of the PFC, which is likely to reduce conscious and deliberate thinking required to maintain the self‐regulatory effort during strenuous exercise. In contrast to the present findings, a higher PFC activation was found during maximal speed walking under extra motivation condition compared to non‐motivational condition (Doren et al. 2024). This contrasting outcome may be due to the different types of exercise protocols with different objectives as the previous study design was to achieve the best record of maximal speed walking, whereas the present study design was to maintain minimum cadence over repeated high‐intensity cycling. While motivation to achieve peak performance appears to increase PFC activation to drive greater neural activity, motivation to sustain repeated and fatiguing exercise tasks is likely to regulate PFC activation for more efficient and economical processing.

Furthermore, this study demonstrated region‐specific differences in PFC oxygenation responses under different motivational conditions during HIIT. Specifically, motivational cues led to significantly lower oxygenation levels in the medial regions of the PFC (OFC and FPPFC) during the later stages of exercise. In contrast, no significant differences were found in the lateral PFC regions (VLPFC and DLPFC) between motivational conditions. These results suggest that motivational cues selectively modulate medial and lateral PFC activity. According to Kouneiher et al. (2009), central regions of the PFC are associated with the monitoring of motivationally salient events, such as rewards and conflict resolution. Conversely, lateral PFC regions are primarily responsible for cognitive control and appropriate behavioural selection via top‐down selection processes. In this system, motivational incentives are conveyed from the medial to lateral regions of the PFC and these functional interactions modulate the balance between immediate and prior information in guiding the cascade of top‐down selection processes occurring in the lateral regions of the PFC. In addition, despite not addressing motivation‐related roles of the PFC directly, a previous study has highlighted distinct functions across PFC sub‐regions, aligning with the finding of the present study (Rodrigo et al. 2014). During a task requiring motor response inhibition, the lateral regions of the PFC (inferior frontal gyrus) showed an increased level of activation relative to a baseline task, whereas medial PFC was significantly deactivated. Using electroencephalography, the effects of motivational audiovisual stimuli on electrical activity in the brain were assessed during a highly fatiguing handgrip‐squeezing task and it was found that such stimuli modulated the frontal and central cortical activities by reducing low‐frequency theta waves and increasing high‐frequency beta waves, respectively (Bigliassi et al. 2016).

Several limitations of this study should be acknowledged. First, only male participants were recruited to control for sex‐based physiological variability, which limits generalisability and highlights the need for future studies to include both sexes. Moreover, a larger sample size and a more comprehensive fNIRS device with additional channels would enhance the accuracy of detecting changes in the variables. While fNIRS is considered suitable for monitoring brain activity during dynamic exercise such as cycling (Menant et al. 2020; Tempest and Reiss 2019), residual motion‐related artefacts cannot be entirely excluded. Nevertheless, consistent experimental conditions and appropriate preprocessing procedures were applied, which likely reduced systematic bias between conditions. In addition, the use of cycling‐based HIIT may not reflect sport specificity, as football is running‐based. However, cycling was selected to facilitate more stable data acquisition, and differences in exercise modality were addressed through individualised HIIT prescription based on PPO, aiming to achieve the same physiological objective as running at maximal aerobic speed to maximise time spent at or near VO_2_max (Buchheit and Laursen 2013; Dall' Agnol et al. 2021). PPO was selected over maximal power output to elicit a higher level of oxygen uptake, compensating for the 7%–18% lower VO_2_max values typically observed in cycle ergometer testing compared to treadmill‐based measures (Millet et al. 2009).

During fitness training, coaches constantly provide motivational cues, particularly during strenuous fitness training sessions, to facilitate the attainment of prescribed exercise intensities and volumes. Such coaching behaviour may be particularly more critical during competitive match play, where players are concurrently exposed to excessive physical and cognitive demands, collectively inducing a state of cortical overload. Under such conditions, the timely delivery of motivational cues by coaches may facilitate the management of perceptual challenges, reduce cortical demands, and consequently delay the impairment in athletic performance. Future research is required to investigate the long‐term effects of high‐intensity fitness training under different motivational conditions on the development of PFC processing efficiency.

## 5 Conclusion

In conclusion, motivational coaching cues such as verbal encouragement and exercise end‐point feedback led to a significantly lower HbO_2_ concentration in the central regions of the PFC (OFC and FPPFC) during the most fatiguing phases of exercise, whereas the lateral regions of the PFC (VLPFC and DLPFC) did not exhibit a significant difference between the conditions at any time interval. These findings highlight that motivational interventions during strenuous exercise may enhance neural efficiency in the central PFC, with these positive effects becoming more pronounced as physiological and perceptual fatigue progressively accumulate. It also supports the region‐specific functions of the PFC associated with motivation during exercise. This study provides new insights into the interaction between motivational coaching cues and repeated high‐intensity exercise, suggesting that lower PFC activation may reflect reduced cognitive load and greater automaticity in motor execution, rather than impaired function. The insights derived from the present findings enable coaches to adapt their coaching behaviours during training or competitive settings in accordance with predefined strategic objectives.

## Funding

The authors have nothing to report.

## Ethics Statement

Institutional ethical approval was obtained for this project at the Institutional Review Board of Seoul National University (IRB approval no. 2412/002–021).

## Consent

Informed consent was obtained from all study participants prior to the start of the experiment.

## Conflicts of Interest

The authors declare no conflicts of interest.

## Supporting information


Supporting Information S1

## Data Availability

The data that support the findings of this study are available from the corresponding author upon reasonable request.
