# Supplementary material for: Motivational Coaching Cues Can Modulate Prefrontal Cortex Activity and Perceptual Responses of Elite Football Players During High‐Intensity Interval Training: A Randomised Crossover fNIRS Study
Source: Eur J Sport Sci. 2026 Jul 30;26(8):e70222. doi: 10.1002/ejsc.70222 (PMC13425583; doi:10.1002/ejsc.70222)
Supplement: Supplementary file 1 — Supporting Information S1 [file EJSC-26-e70222-s001.docx]

**Appendix A: Results of changes in physiological responses to motivational conditions during high-intensity interval training**


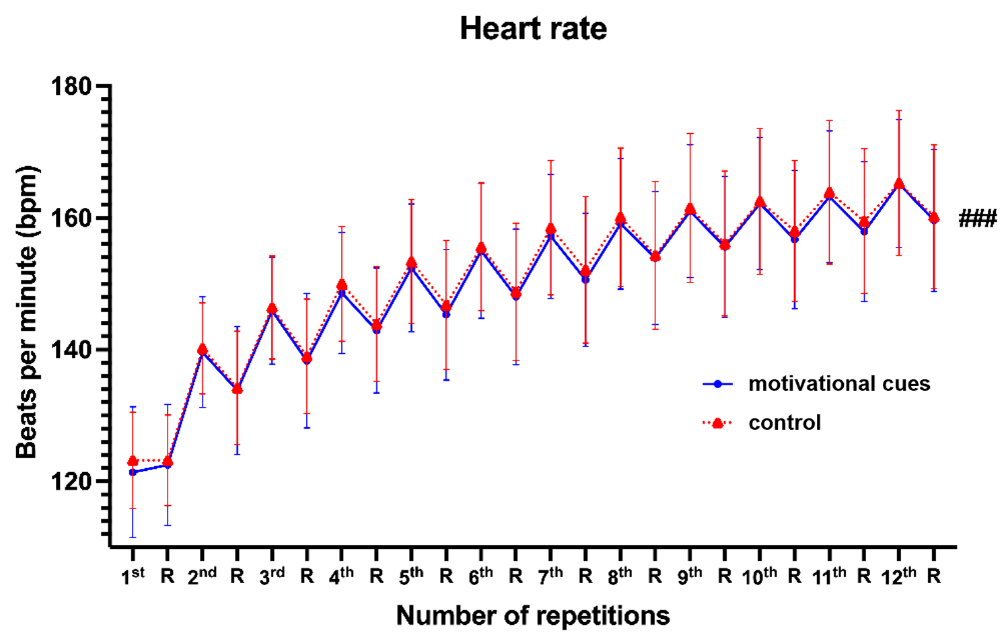


**FIGURE S1 │** Changes in heart rate responses under different motivational conditions over high-intensity interval training. Error bars represent standard deviations.

###p < 0.001 significant increase over time for all conditions.


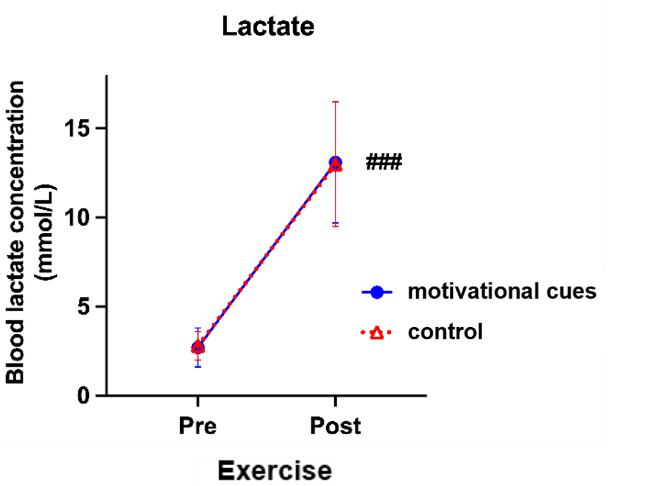


**FIGURE S2 │** Changes in blood lactate concentration under different motivational conditions before and after high-intensity interval training. Error bars represent standard deviations.

###p < 0.001 significant increase over time for all conditions.

**Appendix B: Results of changes in cortical responses to motivational conditions during high-intensity interval training**

**B**

**A**


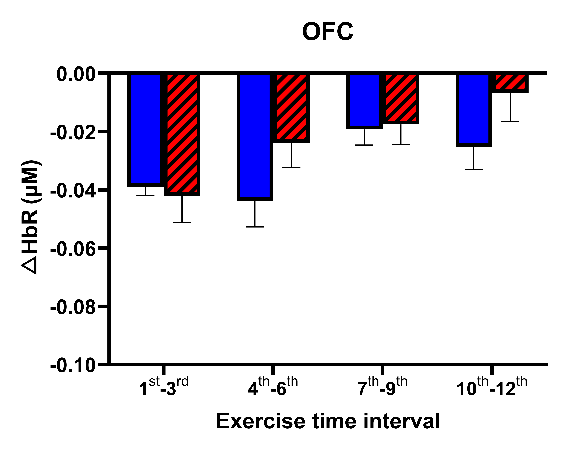

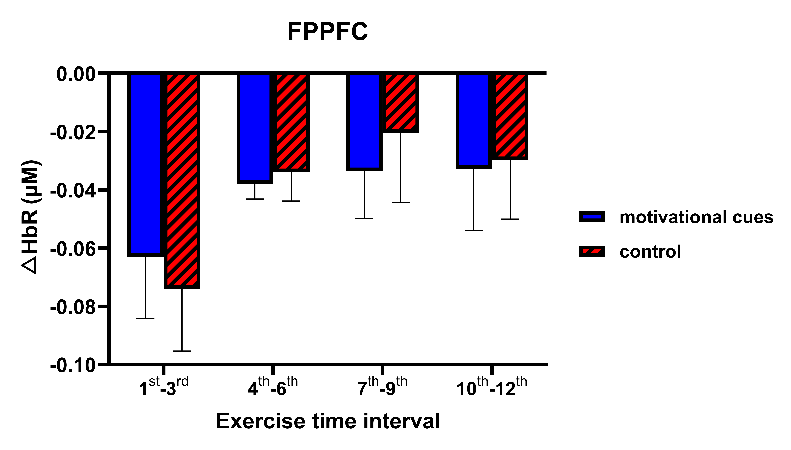


**DD**

**C**


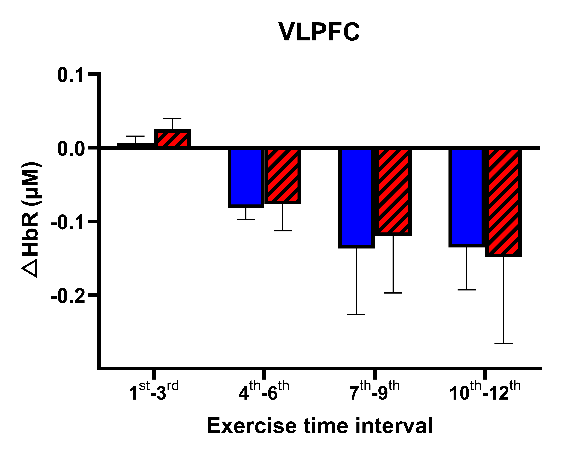

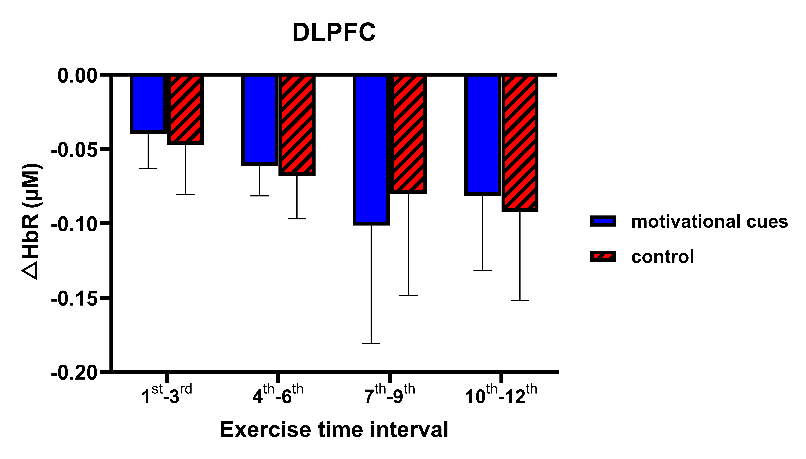


**FIGURE S3 │** Average changes in deoxygenated haemoglobin concentration (△HbR) over four time intervals (1^st^-3^rd^, 4^th^-6^th^, 7^th^-9^th^, and 10^th^-12^th^) according to four regions (OFC, FPPFC, VLPFC, and DLPFC). Error bars represent standard deviations. OFC, orbitofrontal cortex; FPPFC, frontopolar prefrontal cortex; VLPFC, ventrolateral prefrontal cortex; DLPFC, dorsolateral prefrontal cortex.
